# Periprosthetic Osteolysis: Mechanisms, Prevention and Treatment

**DOI:** 10.3390/jcm8122091

**Published:** 2019-12-01

**Authors:** Stuart B. Goodman, Jiri Gallo

**Affiliations:** 1 Department of Orthopaedic Surgery, Stanford University, 450 Broadway St. M/C 6342, Redwood City, CA 94063, USA; 2 Department of Bioengineering, Stanford University, Stanford, CA 94305, USA; 3 Department of Orthopaedics, Faculty of Medicine and Dentistry, Palacky University Olomouc and University Hospital Olomouc, I. P. Pavlova 6, 779 00 Olomouc, Czech Republic; jiri.gallo@volny.cz

**Keywords:** Total joint replacement, total hip arthroplasty, total knee arthroplasty, periprosthetic osteolysis, aseptic loosening, debris-induced inflammation, RANKL-RANK, macrophages, osteoclasts, bisphosphonates

## Abstract

Clinical studies, as well as in vitro and in vivo experiments have demonstrated that byproducts from joint replacements induce an inflammatory reaction that can result in periprosthetic osteolysis (PPOL) and aseptic loosening (AL). Particle-stimulated macrophages and other cells release cytokines, chemokines, and other pro-inflammatory substances that perpetuate chronic inflammation, induce osteoclastic bone resorption and suppress bone formation. Differentiation, maturation, activation, and survival of osteoclasts at the bone–implant interface are under the control of the receptor activator of nuclear factor kappa-Β ligand (RANKL)-dependent pathways, and the transcription factors like nuclear factor κB (NF-κB) and activator protein-1 (AP-1). Mechanical factors such as prosthetic micromotion and oscillations in fluid pressures also contribute to PPOL. The treatment for progressive PPOL is only surgical. In order to mitigate ongoing loss of host bone, a number of non-operative approaches have been proposed. However, except for the use of bisphosphonates in selected cases, none are evidence based. To date, the most successful and effective approach to preventing PPOL is usage of wear-resistant bearing couples in combination with advanced implant designs, reducing the load of metallic and polymer particles. These innovations have significantly decreased the revision rate due to AL and PPOL in the last decade.

## 1. Introduction

Total joint arthroplasty (TJA) is the only fully effective therapeutic choice for patients suffering from end-stage degenerative arthritis. The number of TJAs will gradually increase within the next 20 or 30 years worldwide. The most frequent types of surgery are total hip (THA) and knee arthroplasty (TKA). Survivorship of total hip arthroplasty (THA) has improved, such that 90% of current implants still function optimally at 15 years or more post-operatively [1]. Regardless of the fact that the outcome depends on many variables including the time of THA in service, physical activity of the patient, placement and the type of implant [2], at least 100,000 patients for each million THAs may undergo reoperation within the first 15 years of service. The most frequent late complication of THA is aseptic loosening (AL) accompanied by periprosthetic osteolysis (PPOL), accounting for at least 50% of all THA revisions according to the majority of national registries. Importantly, PPOL can predate AL in THA [3]. Similar or worse survivorship data can be found for TKA [4].

Revision TJA is related to surgical and implant demands as bone defects need to be repaired and joint stability has to be restored. In addition, revision surgery is associated with increased patient morbidity, and great economic burden for the health care system [5,6]. The aim of the review is to summarize current evidence on the mechanisms and treatment of PPOL and AL, in order to provide a rigorous guide for future research and clinical practice.

## 2. Mechanisms Underlying Periprosthetic Osteolysis

### 2.1. The Case of Inflammatory Osteolysis

**Figure 1:**
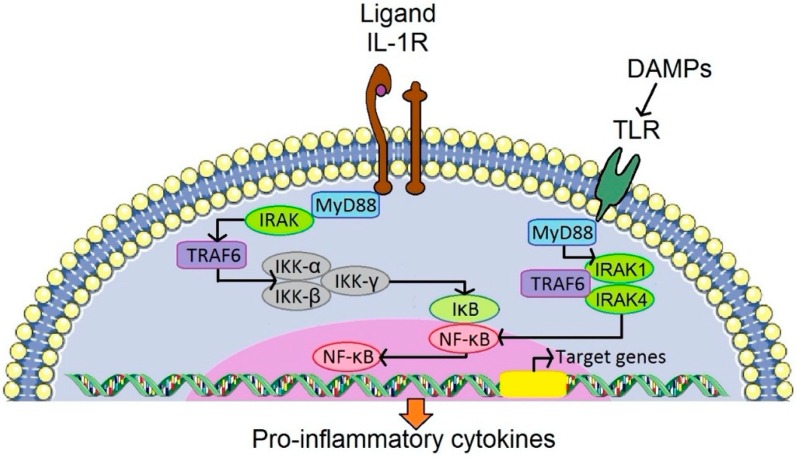
Stimulation of a particular TLR by DAMPs and activation of the IL-1 pathway via stimulation of IL-1R. This results in activation of NF- κB, and leads to synthesis of pro-inflammatory cytokines released into the periprosthetic environment. IL-1α and IL-1β are synthesized as precursor proteins available for their biological action after enzymatic modification. This schema is an example of how the translation of an initial signal (either bacterial, tissue or wear debris origin) into protein synthesis could occur (redrawn according to O’Shea et al., 2019 [26]). DAMP, danger-associated molecular pattern; IκB, inhibitor of κ B; IKK, IκB kinase; IL-1R, interleukin-1 receptor; IRAK, interleukin-1 receptor associated kinase; MyD88, myeloid differentiation primary response 88; NF-κB, nuclear factor κB; TLR, toll-like receptor; TRAF6, tumor necrosis factor receptor-associated factor 6.

**Figure 2:**
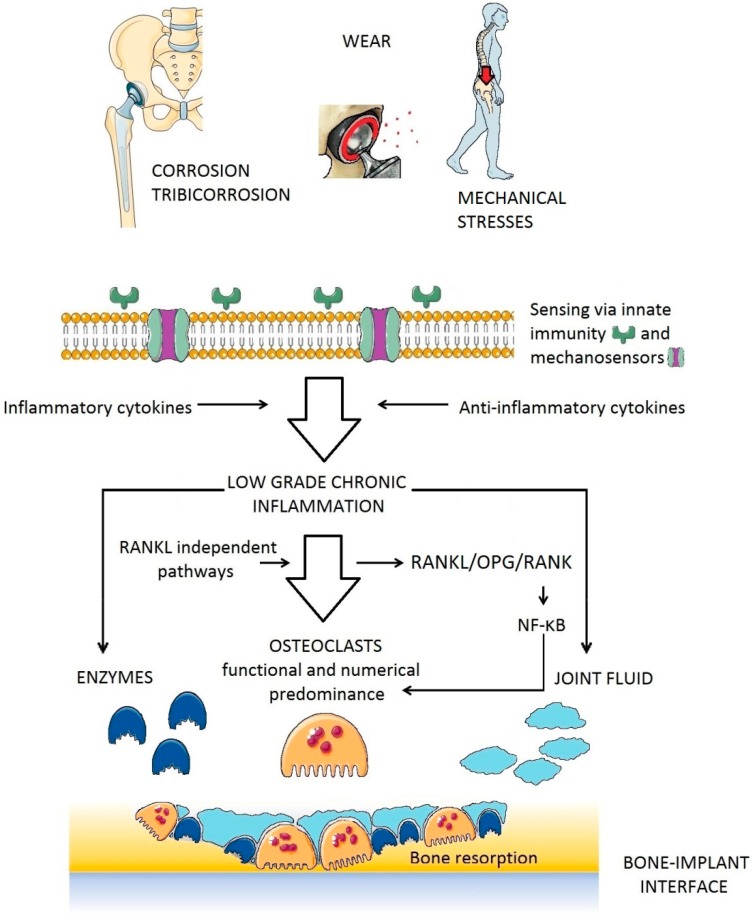
Translation/amplification of signals starting with the interaction of prosthetic byproducts (wear of prosthetic bearings) with cells of the innate immune system and resulting in resorption of the bone in bone multicellular units located along the bone-implant interface. NF-κB, nuclear factor κ B; OPG, osteoprotegerin; RANK, receptor activator of nuclear factor κ B; RANKL, receptor activator of nuclear factor κ Β ligand.

There is strong evidence for the critical role of chronic low-grade inflammation in the mechanism of PPOL [7,8,9]. The evidence comes from in vitro studies [10,11,12,13,14], in vivo experiments [15,16,17,18,19], and analysis of tissues harvested during reoperations [20,21,22,23]. It has been repeatedly demonstrated in vitro and in vivo that after contact between artificially prepared prosthetic byproducts and immune cells, the latter express pro-inflammatory cytokines, chemokines and other substances (Figure 1). The same pro-inflammatory substances have been widely detected in tissues retrieved during reoperations for PPOL and AL [24]. Importantly, the same inflammatory factors are involved in biological processes of the innate immune system, including those dedicated to the immune response to bacterial pathogens. The mechanisms by which cytokines/chemokines are secreted extracellularly have been described in detail [25]. As a result of long-term activation of the inflammatory response around an implant, osteoclasts are activated at the bone–implant interface and initiate bone resorption (Figure 2).

#### 2.1.1. Stimuli of Periprosthetic Inflammation

In general, one can distinguish between wear-related, and non-wear related byproducts. During each step, small particles are liberated from the surface of the softer material by adhesion and abrasion of the bearing surface [27]. With regards to triggering and perpetuation of the intensity of debris-induced inflammation, many parameters play important roles (Table 1).

All lower-limb arthroplasties [28] generate wear particles during each step during the time of service. Ample evidence is available for the pathological role of polyethylene particles in the mechanisms leading to PPOL and AL [11,29,30,31,32]. Biotribological experiments demonstrate that even hard materials like ceramics generate nano-sized debris that could induce inflammation [33].

Corrosive and nano-sized wear byproducts from metallic implants also contribute to the adverse reaction to particulate debris [34,35,36]. At least one research group also reported the inflammatory cell-induced corrosion of TKAs [37]. Metallic debris is of special interest as it might induce late hypersensitivity [38]. Bone cement particles were also examined in relation to debris-induced inflammation [39] and hypersensitivity [40].

However, there is limited knowledge on the interrelations between prosthetic particle size, shape and surface charge and osteoclast differentiation-maturation-survival-functional capacity [20,41,42].

Based on data from new, very sensitive methods for identification of bacterial molecules, bacterial byproducts might contribute to the pathogenesis and/or perpetuation of aseptic PPOL. There is long-term clinical experience demonstrating that prosthetic joint infection is associated with erosive bone resorption if left undiagnosed and untreated. Along these lines, remnants of bacteria circulating in the blood stream might exacerbate inflammation induced by sterile prosthetic byproducts from TJA, despite the absence of clinical infection [43,44]. In principle, the mechanisms which produce bone resorption are similar to those fueled by prosthetic byproducts.

Several studies demonstrate that debris-induced inflammation is more rapid when endotoxin or other proteins specific for bacteria were added to the prosthetic particles [10,17,45]. In addition, some evidence interrelates formation of biofilm on the implant surface with aseptic loosening via chronic inflammation [46]. Some studies suggest that antibiotics could attenuate PPOL and AL [47,48]. On the other side, there is also limited evidence that adding killed bacteria increases bone formation via bacteria induced inflammation [49]. Taken together, further research on this issue needs to be conducted.

Finally, danger-associated molecular patterns (DAMPs) can be powerful stimuli for periprosthetic inflammation via surface cell or intracellular receptors. DAMPs are products of necrotic or stressed cells as a result of long-term ischemia and/or toxic effect of prosthetic debris. Several studies have examined the role of DAMPs in PPOL [50,51,52].

#### 2.1.2. From Stimulation of Receptors of Innate Immunity to Fueling of Inflammation

Inflammation is a universal response of the immune system to external and internal stimuli of danger or non-self, necrotic tissues and adverse mechanical or metabolic stimuli [53]. The intensity of the response could be linked, in part, to similarities between prosthetic particles (especially polyethylene) and bacteria in terms of size and chemical composition [54]. Prosthetic and bacterial byproducts come in contact with a set of innate immunity receptors located on the surface of immune cells and/or intracellularly [55]. The innate immune receptors trigger an acute inflammatory response, resulting in the upregulation and release of inflammatory cytokines, chemokines and reactive oxygen species (ROS). Tumor necrosis factor alpha (TNF, interleukins (IL)-1, 6, 17, and interferon gamma (IF-γ) are considered potent contributors to bone resorption [56,57,58,59]. Some cytokine receptors (e.g., IL-6 receptor) are characterized by tyrosine kinases of the JAK (Janus kinases) family associated with their intracellular domains [60] while for instance IL-1/TLR (Toll-like receptor) is activated via IRAK (interleukin-1 receptor associated kinase) and IL-17 signaling via TRAF2 (tumor necrosis factor receptor-associated factor 2). The details on the cytokine/chemokine network and its functioning are described elsewhere [26,61].

In addition, low-grade inflammation decreases oxygen and nutrients in the affected tissues. Hypoxia could lead to tissue necrosis which could further increase local immunogenicity via the generation of DAMPs [62,63].

Importantly, activated macrophages, similar to neutrophils, produce ROS and nitric oxide (NO) as well as other inflammatory substances; these have been observed in the periprosthetic environment [64,65,66]. ROS has been also shown to contribute to osteoclast differentiation [67].

**Figure 3:**
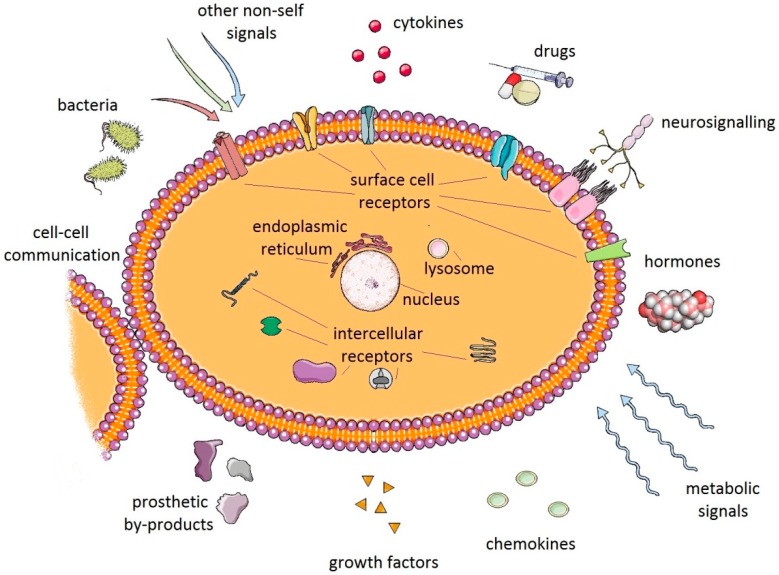
Macrophages and other cells of the innate immune system respond to a myriad of signals emanating from their local environment, including signals resulting from the interaction between prosthetic byproducts and periprosthetic cells. However, it is not known in detail how these interactions translate signals into a specific biologic response of either inflammation or tolerance in a particular patient.

Despite intensive research many aspects of debris-induced inflammation and PPOL remain unexplained. For instance, it is not known how a particular cell or cluster of cells communicates within the complex periprosthetic environment (Figure 3). Little is also known of the role of tolerance in the host tissue response to prosthetic byproducts. Recently, growth and differentiation factor 15 (GDF15) has been revealed as the central mediator of tissue tolerance in response to inflammation [68]. Theoretically, mechanisms of tolerance could also participate in PPOL and AL in patients with TJA. However, further research needs to be conducted.

Cells of innate immunity are capable of sensing wear particles and damaged tissues in the periprosthetic environment. Macrophages are the “conductors” of this reaction [69,70]. M1 polarization of macrophages was observed in association with implant-induced inflammation and necrosis [71,72,73]. There is growing evidence for a role of lymphocytes in the mechanism of AL and PPOL, particularly in relation to hypersensitivity [74,75,76]. Neutrophils are also important in the reaction to wear particles and to microbial loads [77,78]. On the other hand, little is known of the role of mast cells [79,80] and dendritic cells [81,82] in the mechanisms of periprosthetic inflammation and bone resorption.

Non-immunological cell groups like fibroblasts [13], osteoblasts [15], osteocytes [83,84] also contribute to periprosthetic inflammation and osteoclast activity/survival at the bone-implant interface.

#### 2.1.3. Regulation of Debris-Induced Inflammation

Inflammation is tightly regulated in order to prevent excessive collateral damage. The ability of periprosthetic tissues to tolerate a long-term load of prosthetic byproducts seems to be an essential prerequisite for long-term success of TJAs. It may be that PPOL is a particular result of maladaptive inflammation (a driver of bone resorption) compared to an adaptive outcome that normally leads to a stable functional implant. Therefore, the tissues around an implant are tightly regulated in terms of resolution of inflammation, maintaining their cellular, metabolic, and structural health.

A vast body of evidence supports strong and rapid activity of anti-inflammatory mechanisms in response to inflammatory stimulation [85,86]. In addition, tissue homeostatic and regulatory mechanisms are activated in order to prevent excessive tissue damage as a result of inflammation [87]. By these mechanisms, periprosthetic inflammation is strictly regulated.

Some studies show that a number of factors are associated with inflammation-induced mediators of tolerance, for instance via metabolic reprogramming of macrophages. This could modify their plasticity and phenotype including the anti-inflammatory function [88]. A number of cytokines (IL-10, 15), growth factors (TGF-B), and hormones support tissue homeostasis and trigger molecular programs linked to decreasing the activity of periprosthetic inflammation. We are only beginning to understand the role of microRNA in periprosthetic inflammation [89]. Dysregulation of ubiquitin-editing enzyme A20 might also contribute to the development of PPOL and AL as well [90]. Neuroregulation has been linked to implant byproducts-induced chronic inflammation [91]. Semaphorin-3a, neuropilin-1 and plexin-A1 are axonal guidance molecules that have been recently implicated in regulating PPOL [92].

### 2.2. Inflammation-Associated Bone Resorption

**Figure 4:**
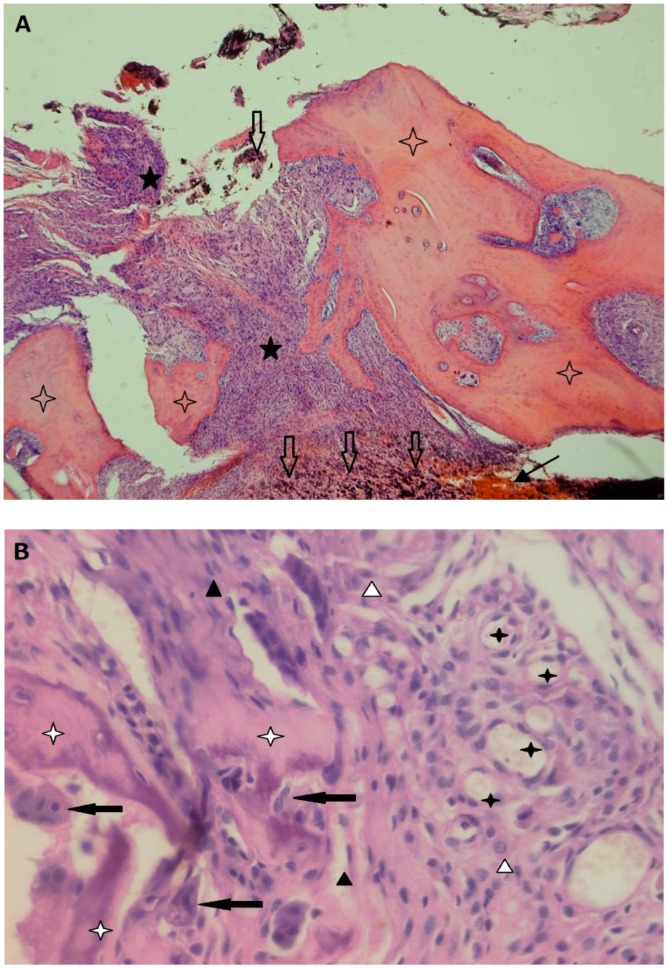
(**A**) An overview of a bone-interface membrane retrieved during a revision of an uncemented THA; bone (blank stars) is interrupted by a fibrous tissue with non-specific granulation tissue (black stars) with dense cellularization (macrophages, fibroblasts, fibrocytes); there is a large number of metallic particles at the bottom (blank arrows) and focus of hemorrhage (black arrow); Hematoxylin and eosin (H&E) staining; 4×. (**B**) Higher magnification showing a number of osteoclasts (black arrows) covering the surface of damaged bone (white stars) surrounded by a fibrous tissue with non-specific granulation tissue containing a predominance of macrophages (white triangles), newly formed capillaries (black stars), spindle fibroblasts and fibrocytes (black triangles). The residual bone surface is covered only by osteoclasts; osteoblasts are not visible; H&E; 200× *(Both photomicrographs courtesy of Department of Clinical and Molecular Pathology, Faculty of Medicine and Dentistry, Palacky University Olomouc; prepared by Zuzana Slobodova, MD).*

There is strong evidence for a major role of osteoclasts in PPOL (Figure 4). Regardless of the particular biological mechanisms leading to osteolysis (whether inflammatory, tumor, or TJA-related), osteoclasts are the only bone-resorbing cells [93,94]. Along these lines, the extent of bone resorption is the function of number, activity, and survival of osteoclasts at a particular place of a bone multicellular unit.

Generally, there are RANK (receptor activator of nuclear factor κ B)-RANKL (receptor activator of nuclear factor κ B ligand)-OPG (osteoprotegerin) dependent and independent pathways leading to osteoclast maturation [95,96,97]. Both of these pathways have been examined in the periprosthetic environment [98,99,100,101]. RANKL stimulates RANK on the surface of osteoclast precursors. RANK activation is followed by further biochemical steps involving adaptor molecules such as tumor necrosis factor receptor-associated factor 6 (TRAF6), which leads to the activation of mitogen-activated protein kinases (MAPKs), and the transcription factors NF-κB (nuclear factor κ B) and AP-1 (activator protein-1). Activated NF-κB influences osteoclast differentiation, activation, and survival via the nuclear factor of activated T-cells cytoplasmic 1 (NFATc1), which is the key osteoclastogenesis regulator [102,103].

Bone consists of a collagen-rich, mineralized extracellular matrix that has to be cleaved by metalloproteinases to cause bone resorption. Bone marrow derived stromal cells express metalloproteinases in response to multiple signaling pathways [104]. A wide range of metalloproteinases participate in the induction and propagation of the osteolytic effect [105]. Distortion of the balance between bone metalloproteinases and their tissue inhibitors is also a result of chronic inflammation. Specific matrix metalloproteinases can be overexpressed at the bone-implant interface, contributing to the expansion of PPOL in this way [106,107]. In addition, elevated ROS can impair proteasome function contributing to PPOL and AL [108,109]. Cathepsin K, a papain-like cysteine protease member of the cathepsin family of proteases that is synthesized by osteoclasts into their sealing zones also plays a prominent role in bone resorption [110]. It can degrade type I collagen as well as other organic structures within the bone matrix.

### 2.3. The Implant–Bone Interface: A Series of Bone Multicellular Units

Although much research concerning the periprosthetic microenvironment has focused on wear particle disease, inflammation and the degradative processes of bone, byproducts from implants also have an effect on bone formation and remodeling. The bone–implant interface is essentially a series of bone multicellular units (BMUs) which are composed of osteoclasts, osteoblasts, and other cells of the MSC (mesenchymal stem cell)-osteoblast lineage [111]. Although the majority of research on PPOL has concentrated on cells of the monocyte/macrophage/foreign body giant cell/osteoclast lineage, other cells in the BMU, as well as fibroblasts, vascular progenitors and others play prominent roles in the development of PPOL. Previous research has documented adverse effects of different byproducts of wear on osteoblasts [15,84,106,112,113,114], osteocytes [83,84,115], and mesenchymal stem cells and osteoblast progenitors [116,117,118]. In general, chronic inflammation associated with byproducts of wear suppresses bone formation by inhibiting the proliferation, differentiation, maturation and function of progenitor cells and their downstream lineage cells. These deleterious effects result in dysregulation between bone formation and resorption, tipping the scales towards osteolysis. Ongoing inflammation also favors areas of cell necrosis and fibrosis, undermining the stability of the prosthesis and its ability to withstand physiological loads.

### 2.4. Mechanical Osteolysis and Synergies with Inflammatory Agents

Mechanical loading is a major anabolic stimulus for bone formation [119]; conversely, stress-shielding associated with specific implant designs, their modulus of elasticity and other factors can contribute to bone loss around TJA [120,121]. Every joint is subject to mechanical forces and loads and, therefore, the prosthetic joint serves as a biological tool for transmission of mechanical forces. A periarticular bone is designed to accept high loads occurring repetitively during gait, running and other activities.

An unstable implant can induce gradual bone resorption similar to when mechanical movement at the site of a fracture contributes to the development of non-union associated with bone resorption [122,123,124]. Some studies have demonstrated that mechanical forces generated either by joint fluid or the implant surface can also lead to bone resorption [115,125,126,127,128,129]. The mechanisms by which mechanical forces contribute to PPOL have not been fully understood to date. Directly transmitted forces are closely associated with pressure-induced bone resorption; indirect forces may work via inflammatory processes [130] and modulation of osteoclastogenesis [131]. Innate immune cells [130], fibroblasts [132], osteocytes [115] and other cells can sense mechanical stimuli and respond to physiological and non-physiological forces or pressures.

Based on the above, one may conclude that there is relatively strong evidence for the role of mechanical forces and pressures in PPOL. In addition, these mechanical pressures and forces can interact with biological processes of PPOL [133]. Finally, some believe that mechanical forces are the primary causative mechanism while the inflammatory pathways are secondary to mechanical loosening [134]. In summary, regardless of an essential role of mechanical loading on periprosthetic bone structure, the evidence for inflammatory PPOL is stronger [3].

## 3. Treatments for Periprosthetic Osteolysis

### 3.1. Operative Treatment

Traditionally, osteolysis associated with wear particles and byproducts from TJA is seen as a “surgical disease” [135]. In other words, once osteolysis is observed radiographically, wear of the joint couple will continue and therefore PPOL will inevitably progress; these events will jeopardize the longevity of the TJA and surrounding bone and soft tissues. Surgery is indicated to correct the failing articulation and address prior and ongoing potential bone loss. Although the surgical aspects of revision surgery are not the focus of this article, a brief description of the surgical strategy will be presented. The strategies outlined below are for THA with PPOL; although the philosophy is similar, the approaches for PPOL in other joints will vary depending on the anatomical location, structure, design, function and materials used.

If the prosthesis is stable (well fixed), well aligned, functional, and suitable modular replacement parts can be obtained, then revision surgery is limited to revising the bearing surfaces only and possibly grafting of bone either by bone allografts, or byproducts of bone (such as demineralized bone matrix, iliac crest concentrated cell aspirate), bone-graft substitutes (such as the various osteoconductive or osteoinductive materials), biologics (such as BMP-2, bone morphogenetic protein-2), or combinations thereof [136]. There is controversy regarding whether the outcome for partial revision of modular parts (e.g., polyethylene liner/head exchange only) is better than revision of the entire component (e.g., the polyethylene liner and acetabular metal shell of hip replacements). However, when the entire component is revised, there is additional surgically induced bone loss as well as that due to the chronic inflammatory reaction to the wear byproducts. Thus, if outcomes are equal, the simplest route, i.e., liner/head exchange only, is often preferred to revision of the entire component. Once the “wear generator” has been corrected by revision surgery, the osteolytic areas may undergo ossification or stop their progression [136].

Operations on the femoral side of THAs that demonstrate osteolysis are often more challenging [135,137]. Cemented components may show cement cracks and fragmentation, leading to substantial endosteal osteolysis and even pathologic fracture. These cases are revised, often using an extended femoral osteotomy to excise all of the debris and well-fixed acrylic cement, prior to re-implantation of a longer stemmed component (often cementless). Well-fixed cementless implants may demonstrate proximal femoral osteolysis due to the biological reaction to polyethylene debris in the effective joint space [138]. Dealing with the bearing surface is the first priority. If the femoral stem is stable, bone grafting of the proximal femur with crushed allograft croutons (for contained defects) or strut grafting (uncontained defects) may be indicated. If the prosthesis is undermined to the point that the prosthesis or bone might fracture or become loose, then revision surgery is indicated, together with reconstitution of lost bone via strut and cancellous bone grafting [139]. If the proximal femoral bone stock cannot support a conventional prosthesis, then a tumor prosthesis, or a structural femoral allograft-prosthetic composite may be necessary [139].

Byproducts from metal-on-metal implants and conditions in which metal corrosion has occurred at modular junctions may lead to osteolysis and even large pseudotumors, compromising bone and soft tissue. These cases need thorough investigation and surgical treatment [140]. The osteolytic area and pseudotumor are debrided and modular metal-on-metal bearings are exchanged for other ones, usually ceramic-on-polyethylene. Corrosion of modular junctions may necessitate exchange of bearing surfaces (e.g., ceramic head substituted for a cobalt chrome alloy one in THA), or even excision of an entire, well fixed component if there is major compromise of the structural integrity of femoral neck.

Osteolytic areas around knee and shoulder arthroplasties are treated using a similar philosophy as for THA [141].

### 3.2. Non-Operative Treatment

There are no long-term studies that demonstrate the efficacy of non-operative pharmacologic treatment for PPOL. Although in vitro and in vivo studies may show limited short-term mitigation of osteolysis, wear particle-associated osteolysis in humans is progressive, and my lead to alternative forms of wear; this may occur when the polyethylene liner is completely worn through, and the metallic femoral head now articulates with a metallic shell. This latter scenario is particularly disastrous because the result is multiple types of wear particles, and an articulation that must be excised rather than salvaged in part. As a result, a place for a particular non-operative intervention should be much earlier before gross damage of bearings is detected. The aim of such intervention is to limit the size of bone defects as well as postpone the need for reoperation. In addition, this strategy might be beneficial for patients susceptible to PPOL who develop PPOL earlier and perhaps under lower loads of prosthetic byproducts. Finally, non-operative intervention may be a choice for patients who cannot undergo re-operation immediately, with the hope of diminishing the extension of bone defects. However, a benefit of all the above potential indications for non-operative treatment must be demonstrated [142].

Traditionally, pharmacologic therapies are classified into those that affect bone degradation, and those that affect bone formation. This classification is misleading because bone formation and degradation are linked, and more often than not, therapies affect both arms of the process of bone modeling. Thus, the following will review some of the more common treatments for osteolysis that are or could become clinically relevant in humans [143,144].

#### 3.2.1. Bisphosphonates

Bisphosphonates are medications taken orally or parenterally for the treatment of osteoporosis, and osteolysis associated with metabolic and metastatic bone disease. These medications are synthetic analogues of pyrophosphate, and have been used in the treatment of PPOL in an “off label” or “physician directed” manner [145]. The mechanism of action is primarily on the osteoclast, which undergoes apoptosis, thus inhibiting bone resorption, although there are also downstream effects on osteoblast function. These medications do not affect the pro-inflammatory profile of periprosthetic tissues, at least in the short term [146]. Whereas there may be some utility of bisphosphonates in mitigating adverse mechanically-based bone remodeling associated with implantation of a prosthesis, the use of bisphosphonates has not garnered much support for the treatment of particle-associated PPOL [147]. To the authors’ knowledge, there have been no instances of periprosthetic fragility fractures associated with the use of bisphosphonates for PPOL.

#### 3.2.2. Other Treatments Aimed at Osteoclasts

RANKL is a protein released from osteoblasts and other cells, and activates the transmembrane protein RANK on the surface of osteoclasts. RANK regulates osteoclast activity and subsequent bone resorption [148]. Denosumab is a human monoclonal antibody to RANKL that blocks the binding of RANKL to RANK, thereby inhibiting osteoclast activity and function [149]. Other inhibitors of osteoclast activity such as Cathepsin K activity have also been developed. Although a human trial may be underway using Denosumab, there is no report yet of its efficacy in the scenario of particle-induced osteolysis in humans [142].

#### 3.2.3. Treatments Aimed at Specific Pro-Inflammatory Cytokines

Substances released from macrophages and other cells stimulate PPOL by increasing bone resorption by cells of the macrophage-foreign body giant cell-osteoclast lineage, and decreasing the proliferation, differentiation and function of cells of the MSC-osteoblast lineage [7,150,151,152,153]. Indeed, the macrophage is the orchestrator of these events [70]. The mechanisms by which macrophages and other cells accomplish osteolysis is via the release of pro-inflammatory cytokines, chemokines, reactive oxygen species, and other substances under the umbrella of the innate immune system [59,151]. The inflammatory processes associated with innate immunity are generally non-specific to the particular stimulus, but there also appears to be a memory component to the innate immune system [154]. Furthermore, the system is overly redundant so that inhibition of one cytokine pathway does not dramatically affect the overall consequences of injury or other adverse stimuli. Although in vitro and animal studies have been impressive in limiting or reversing the bone resorption due to different wear particles [143,145], in vivo studies in humans have not been pursued due to the complexity of the inflammatory pathways and potential adverse effects of interfering with inflammation, namely infection, inhibition of bone formation etc.

#### 3.2.4. Treatments Targeted to Events More Upstream from the Inflammatory Cytokines

PPOL is the biological result of a wear particle-induced microenvironment in which pro-inflammatory cytokines, chemokines, reactive oxygen species and other substances stimulate cells to degrade bone and inhibit the formative processes of bone matrix [101,150,151,155]. Due to the overall redundancy of the inflammatory process [145], and the ineffectiveness of inhibiting single cytokines, treatments have been proposed to mitigate osteolysis more upstream. These include interfering with the key proinflammatory transcription factor: nuclear factor kappa B (NF-κB), altering the polarization state of macrophages from a pro- to an anti-inflammatory phenotype and inhibiting local macrophage trafficking [144]. The first approach, i.e., inhibiting NF-κB, has been reported in vitro and in vivo in animals [156,157,158,159,160,161]. Toll like receptors and mitogen-activated protein (MAP) kinases also appear to be important pathways in this cascade [155,162,163]. It may be difficult to translate these strategies to humans, except perhaps for local delivery.

Altering macrophage phenotype from an M1 pro-inflammatory to an M2 anti-inflammatory particle-induced inflammation has been accomplished in vitro and in vivo [50,164,165]. This approach would best be accomplished using local methods of delivery.

Migration of macrophage to sites of inflammation is due to the liberation of specific chemokines in response to local environmental cues. In vitro and in vivo animal models have established that depletion of macrophages, or inhibition of important macrophage pathways such as the MCP-1 (monocyte chemoattractant protein-1)-CCR2 (C-C chemokine receptor 2) axis can inhibit systemic trafficking and osteolysis [164,166,167,168,169].

#### 3.2.5. Anabolic Treatments

Various different strategies have been considered for treating the anabolic side of particle-associated osteolysis [145].

The 1-34 amino acid portion (1-34) of parathyroid hormone (PTH) is an anabolic substance that increases bone formation via PKA (protein kinase A) and Wnt/β-catenin pathways, and also increases the section of OPG, an antagonist of RANK. This medication is given intermittently and approved for use in the treatment of post-menopausal osteoporosis. This treatment might mitigate, particle-induced osteolysis

Recombinant OPG gene therapy has also been shown to inhibit osteolysis in an animal model, however the adverse effects of this strategy are not immediately translational [170].

Icariin has been shown to protect osteolysis by promoting osteogenic differentiation of MSCs via activation of the Wnt/β-catenin signaling pathway [171]. This flavonoid also inhibits the formation and activation of osteoclasts. Other medications such as melatonin have shown similar results [172,173]. Strontium ranelate is another potential treatment for particle-induced osteolysis. This drug is currently approved for the treatment of post-menopausal osteoporosis. Strontium ranelate stimulates proliferation of pre-osteoblasts, suppresses osteoclastic differentiation and increases osteoclastic apoptosis [174].

#### 3.2.6. Cell Therapy

Local therapeutic delivery of cells can directly or indirectly affect osteolysis. Autologous bone grafting is a form of local cell therapy, in that osteoblasts and other cells in the bone graft composite can engraft, modulate the inflammatory cascade and provide autocrine and paracrine factors to support bone healing. Crosstalk between macrophages and MSCs is an ongoing process in all inflammatory bone disorders and in bone healing [175]. Indeed, local delivery of MSCs is a putative treatment for acute and chronic inflammation in many organ systems. Our group (SBG) has used this approach in vitro and in vivo to modulate inflammatory reactions and facilitate bone healing [176,177]. We have developed a pre-conditioning protocol for MSCs using short-term exposure of MSCs to TNFα and lipopolysaccharide (LPS) to enhance immunomodulation (by promoting polarization to an anti-inflammatory macrophage phenotype) and osteogenesis [178]. In addition, training MSCs by exposure to inflammatory cytokines and other substances appears to impart an innate immune memory of the prior stimulus [179]. We have also developed NF-κB sensing and IL-4 over-expressing genetically modified MSCs to treat chronic inflammation [176,180]. Local delivery of these modified MSCs may have a future role in the treatment of PPOL.

## 4. Conclusions

Prosthetic byproducts have a major effect on biological parameters in the periprosthetic environment. Key check points potentially applicable to modification of implant-induced responses are those associated with (i) macrophage polarization; (ii) pro-osteoclastic signaling of periprosthetic cells; (iii) the RANKL-mediated osteoclastogenesis; (iv) the NF-κB and mitogen-activated protein kinase pathways involved in inflammation/osteoclastogenesis; (v) extension of necrosis vs. apoptosis in the periprosthetic membranes. Mechanical factors such as repetitive stress/strain could also contribute to PPOL, however, these are difficult to modify.

Taken together, a detailed knowledge of the pathophysiology of PPOL suggests new possibilities for preventative measures that could dramatically decrease the rate of PPOL and AL of TJA in the future. These principles could be applied simultaneously or successively in order to achieve a synergistic effect. Various non-operative therapeutic interventions for the treatment of PPOL are still in the preclinical stage of assessment. Some have entered limited clinical trials. However, it must be kept in mind that progressive PPOL due to wear particles is not only a biological phenomenon, but also a material issue, indicating failure of the bearing surface or modular interfaces. In this respect, at this point in time, there are no evidence-based non-operative treatments for this disease in clinical practice. Conversely, we have strong data supporting the use of modern wear-resistant bearings and, thus, decreasing the rate and size of PPOL. This data is the strongest evidence supporting the concept that byproducts of prostheses lead to PPOL.

## Figures and Tables

**Table 1:** List of parameters potentially influencing the induction and perpetuation of debris-induced inflammation leading eventually to PPOL.

Parameter	Potential Role for	Estimated Evidence
**Particle size** *(Particles between 0.1 and 1 µm seem to be the most effective ones)*	Induction/perpetuation of inflammation	Strong
**Origin of particles** *(Polymers such as polyethylene and PMMA, and metal particles are the most inflammatory)*	Intensity of inflammation; type of inflammation; induction of hypersensitivity	Strong
**Particle shape** *(Irregular shaped particles with a rough surface topography are more inflammatory)*	Intensity of inflammation; type of inflammation	Weak
**Particle amount** *(A threshold for PPOL has been determined)*	Induction/perpetuation/intensity	Strong
**Particle surface charge** *(Need to be verified)*	Intensity of inflammation; type of inflammation	Weak
**Clearing capacity of periprosthetic tissues**	Intensity of inflammation; type of inflammation	Moderate
**Anti-inflammatory mechanisms**	Regulate intensity of inflammation in periprosthetic tissues	Strong
**Individual susceptibility** *(genetic make-up* e.g., *SNPs may predispose to a more aggressive inflammatory reaction)*	Intensity of inflammation; type of inflammation; tissue homeostasis; osteoclast survival/activity	Moderate

PMMA, polymethylmethacrylate; PPOL, periprosthetic osteolysis; SNP, single-nucleotide polymorphism. Strong evidence means that many studies have demonstrated consistently the effect; moderate evidence means that the effect has been demonstrated in several studies but there is some doubt about their consistency or strength; weak evidence implies that one or two studies show the effect of a parameter or the link between a parameter and an effect can be translated from other fields of bone biology/immunology.
